# Effect of music listening on perioperative anxiety, acute pain and pain catastrophizing in women undergoing elective cesarean delivery: a randomized controlled trial

**DOI:** 10.1186/s12871-023-02060-w

**Published:** 2023-04-03

**Authors:** Avinash Kakde, Ming Jian Lim, Haiying Shen, Hon Sen Tan, Chin Wen Tan, Rehena Sultana, Ban Leong Sng

**Affiliations:** 1 Department of Women’s Anesthesia, KK Women’s and Children’s Hospital, 100 Bukit Timah Road, Singapore, 229899 Singapore; 2 Division of Nursing, KK Women’s and Children’s Hospital, Singapore, Singapore; 3 Anesthesiology and Perioperative Sciences Academic Clinical Program, Duke-NUS Medical School, Singapore, Singapore; 4 Center for Quantitative Medicine, Duke-NUS Medical School, Singapore, Singapore

**Keywords:** Analgesia, Catastrophizing, Obstetrics, Pain

## Abstract

**Background:**

Anxiety may adversely impact mother and her newborn. Music listening is a safe and efficacious treatment that may to reduce perioperative anxiety. The effect on acute pain and pain catastrophizing scores remains unclear. We aimed to determine whether perioperative music listening reduces anxiety, acute pain, and pain catastrophizing scale (PCS) scores following elective cesarean delivery under spinal anesthesia.

**Methods:**

After randomization into music listening and control groups, baseline patient characteristics, visual analog scale-anxiety (VAS-A) scores, pain scores, PCS total and sub-scores, and music preferences were collected preoperatively. Before surgery, parturients in the experimental group listened to music of their own choice for 30 min. Music listening was continued during administration of spinal anesthesia and cesarean delivery, and for 30 min following surgery. Postoperative VAS-A score, acute pain score, PCS scores, music preferences, satisfaction score, and feedback were recorded.

**Results:**

We analyzed 108 parturients (music: n = 53; control: n = 55). Music listening was associated with reduced postoperative VAS-A (mean difference (MD) -1.43, 95%CI -0.63 to -2.22), PCS total score (MD -6.39, 95%CI -2.11 to -10.66), PCS sub-scores on rumination (MD -1.68, 95%CI -0.12 to -3.25), magnification (MD -1.53, 95%CI -0.45 to -2.62), and helplessness (MD -3.17, 95%CI -1.29 to -5.06) sub-scores. There was no significant difference in postoperative acute pain scores. The majority (> 95%) of parturients reported “excellent” and “good” satisfaction with music listening, and most provided positive feedback.

**Conclusion:**

Perioperative music listening was associated with reduced postoperative anxiety and lower pain catastrophizing. Based on the good patient satisfaction and positive feedback received, the use of music listening in the obstetric setting is recommended.

**Trial registration:**

This study was registered on Clinicaltrials.gov NCT03415620 on 30/01/2018.

## Introduction

Approximately 25% of parturients reported experiencing anxiety during pregnancy, which is associated with increased risk of maternal complications e.g., pre-eclampsia and emergency cesarean delivery [1, 2]. Additionally, parturients with pre-existing anxiety had significantly higher pain scores following cesarean delivery, which may in turn increase their risk of developing persistent pain and postpartum depression [3, 4]. Although perioperative anxiety and pain are often treated pharmacologically, concerns of fetal or breastmilk transfer and risk of adverse effects often limit peripartum use.

Recently, music listening has emerged as a safe and efficacious treatment modality to reduce perioperative anxiety associated with cesarean delivery. A meta-analysis of 15 studies reported that music listening reduced intraoperative anxiety and postoperative opioid consumption following cesarean delivery [5]. However, most of the available studies involved parturients in Europe, USA, and Iran, with limited evidence of the use of music listening in cesarean settings in Southeast Asia [5]. In Singapore, the efficacy of music listening in reducing anxiety and acute pain has been reported in orthopedic or gynecological surgeries, but no data is available in the context of cesarean delivery [6–8].

Interestingly, patients undergoing total joint arthroplasty with a high preoperative anxiety had significantly higher pain catastrophizing status and higher postoperative pain [9]. Women undergoing hysterectomy further demonstrated that pain catastrophizing may act as a mediator between preoperative anxiety and postoperative pain intensity [10]. Other studies have suggested that pain catastrophizing is associated with greater acute pain intensity and increased risk of developing post-cesarean persistent pain [11–13]. However, it is unknown whether music listening could reduce postoperative pain catastrophizing in parturients after cesarean delivery.

Hence, we investigated whether perioperative music listening has significant impact on anxiety scores (primary outcome), acute pain scores, and Pain Catastrophizing Scale (PCS) scores following elective cesarean delivery. We hypothesized that parturients receiving perioperative music listening would have significantly reduced postoperative anxiety, acute pain, and pain catastrophizing compared to parturients who did not receive music listening.

## Methods

This randomized controlled trial adheres to the applicable Consolidated Standards of Reporting Trials (CONSORT) guidelines and was approved by the SingHealth Centralized Institutional Review Board (2017/2930) on 27/10/2017 and registered in Clinicaltrials.gov (NCT03415620) on 30/01/2018. We included healthy parturients aged 21 to 50 years old with American Society of Anesthesiologists (ASA) physical status 2, and undergoing elective cesarean delivery under spinal anesthesia at KK Women’s and Children’s Hospital, a specialist obstetric center in Singapore. Parturients with hearing impairment, significant respiratory disease and obstructive sleep apnea, conversion to general anesthesia due to surgical/ anesthesia complication/wearing off spinal anesthesia effect due to prolonged surgery timing, patients with placenta previa and placenta accreta pathology and who were unable to understand the pain and psychological assessment questionnaires were excluded.

### Intervention

Enrolled parturients were randomized in 1:1 allocation ratio to experimental (music listening) and control (no music listening) groups. The allocation sequence was created by the study statistician (RS) using a computer-generated random number generator and concealed using sequentially numbered opaque sealed envelopes. Preoperatively, a study investigator would open an envelope containing the group allocation of recruited parturient. Parturients in the experimental group were asked to listen to music of their own choice streamed from Apple Music via earphones connected to an Apple device (either iPod Touch or iPhone) for approximately 30 min. During administration of spinal anesthesia and cesarean delivery, music listening was continued via a portable Bluetooth speaker to enable verbal communication with the anesthetic and surgical teams. Following the surgery, parturients continued listening to their chosen music for approximately 30 min via earphones in the post-anesthesia care unit (PACU).

#### Cesarean delivery and data collection

All parturients received single-shot spinal anesthesia with 0.5% hyperbaric bupivacaine, morphine 100 µg of, and fentanyl 15 µg. After fetal delivery, uterotonics and antiemetics were administered as per routine institutional practice. Baseline patient characteristics, visual analog scale – anxiety (VAS-A) scores, pain scores (0: no pain, 10: worst pain imaginable), PCS total and sub-scores, and music preferences were collected preoperatively. We also recorded pain scores relating to local anesthetic injection for spinal anesthesia, as it was shown to predict acute pain after cesarean delivery [14]. Intraoperative hemodynamic in terms of baseline systolic blood pressure, maximum and minimum heart rate, maximum and minimum systolic blood pressure, and maximum and minimum diastolic blood pressure were collected intraoperatively. Also, the duration of surgery, additional intraoperative analgesia/ medication and any anesthesia/surgical complications were documented. In the PACU, VAS-A scores, acute pain scores, postoperative PCS total and sub-scores, and music preferences were collected. Satisfaction scores with preoperative and postoperative music listening were also assessed. Those patients who complained of pain in PACU (VAS score of > 5) received intravenous paracetamol 1 gram and/or other analgesic medications as rescue analgesia at the discretion of the treating anesthesiologist.

### Statistical analysis

Our primary aim was to determine the difference between postoperative anxiety levels in terms of VAS-A scores between the two groups. A sample size of 92 (46 in each group) provided 85% power, with the following assumptions: mean difference (MD) of postoperative VAS-A between control and experimental groups of 2.1 (standard deviation (SD) 3.3), allocation ratio 1:1, and level of significance of 5% [15, 16]. Sample size was calculated using a two-sided, two-sample, equal-variance t-test. After accounting for 16% withdrawal after randomization, a total of 110 (55 in each group) parturients was required. Our secondary aims were to determine the difference in postoperative acute pain score, PCS total score and its sub-scores. We reported satisfaction scores and music preferences for parturients who received music listening. All variables were summarized based on group allocation (experimental group/ control group). Categorical and continuous variables were summarized as frequency (proportion) and mean (SD), respectively. Differences between the groups were tested using two-sample t-tests and Chi-square tests for continuous and categorical variables, respectively. All tests were two-sided and p-values < 0.05 were considered statistically significant. All analyses were conducted using SAS 9.4© (2016) by SAS Institute Inc., Cary, NC, USA.

## Results

**Fig. 1:**
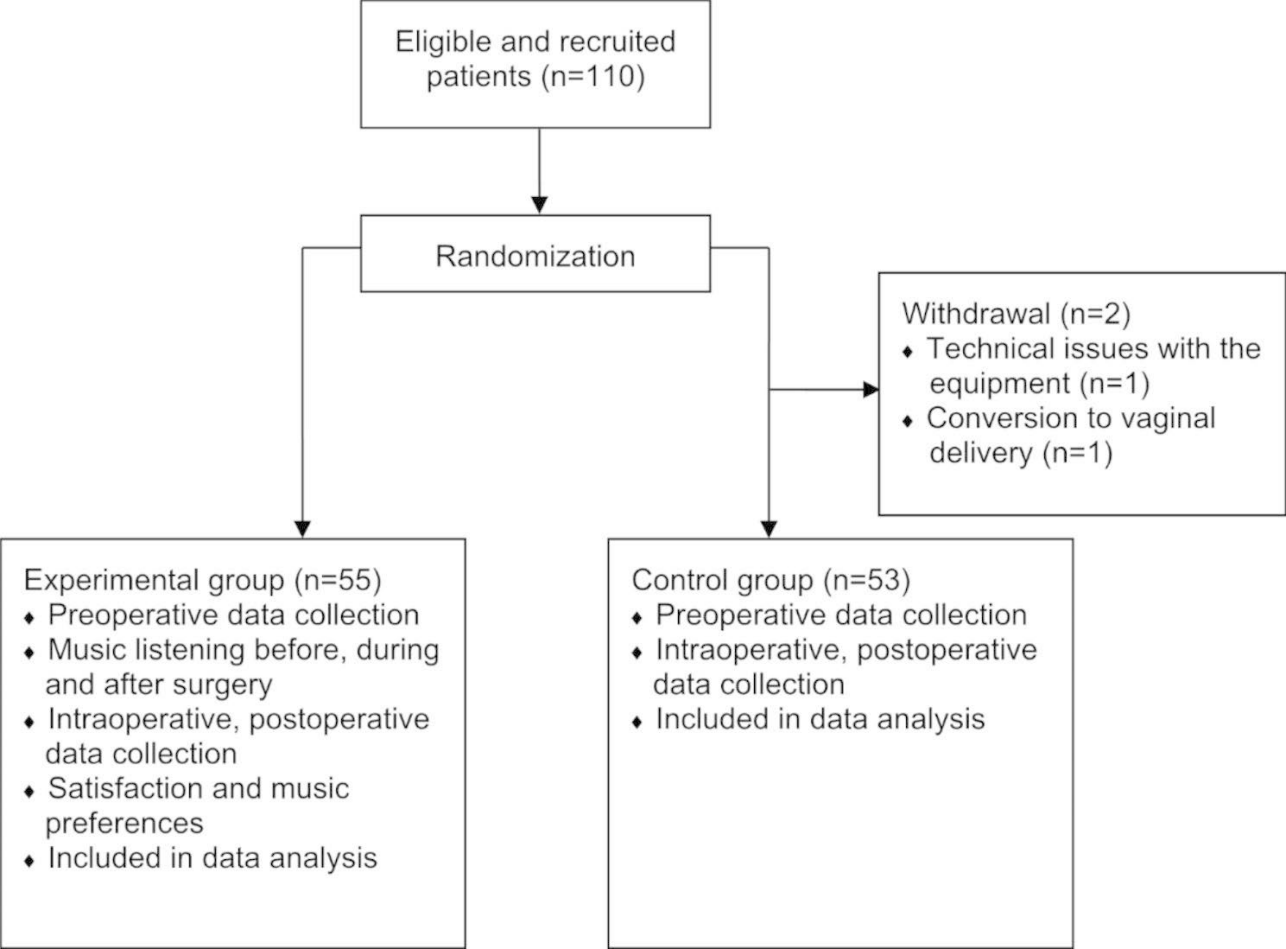
Study flow diagram

A total of 110 parturients undergoing cesarean delivery between May and Sep 2022 were randomized in 1:1 allocation ratio of experimental (music listening) and control (no music listening) groups (Fig. 1). Two parturients withdrew from the study due to technical issues with the equipment and conversion to vaginal delivery. Hence, data from 108 parturients (experimental: n = 53; control: n = 55) were analyzed. Baseline and clinical characteristics are summarized in Table 1, whereby no significant difference was found in these characteristics between experimental and control groups. No anesthetic/ surgical complication was reported in all recruited parturients, except for one patient from control group who had intraoperative transient vasovagal syncope, and one patient from control group having postoperative drug allergy. None of the patient required intraoperative rescue analgesia. All the patients in the experimental group listened to music during the preoperative and intraoperative period. However, five patients from the experimental group chose to rest in the postoperative period and not to listen to music.

**Table 1:** Baseline and clinical characteristics

Variable	Music group (n = 53)	Control group (n = 55)	Total (n = 108)
Age (years), mean (SD)	33.6 (4.6)	34.4 (4.3)	34.0 (4.4)
Race, n (%)			
Chinese Malay Indian Others	25 (47.2) 12 (22.6) 7 (13.2) 9 (17.0)	28 (50.9) 16 (29.1) 8 (14.5) 3 (5.5)	53 (49.1) 28 (25.9) 15 (13.9) 12 (11.1)
BMI (kg.m^− 2^), mean (SD)	29.6 (4.0)	29.2 (4.2)	29.4 (4.1)
Gestational age (weeks), mean (SD)	38.3 (1.0)	38.4 (0.7)	38.4 (0.9)
Baseline systolic blood pressure (mmHg), mean (SD)	125.8 (15.4)	129.9 (20.5)	127.9 (18.2)
Pain score upon LA injection (0 to 10), median (IQR)	4.0 (3.0)	5.0 (3.0)	4.0 (3.0)
Maximum systolic blood pressure (mmHg), mean (SD)	136.3 (16.3)	140.3 (17.9)	138.4 (17.2)
Minimum systolic blood pressure (mmHg), mean (SD)	92.3 (13.8)	92.6 (16.0)	92.5 (14.9)
Maximum diastolic blood pressure (mmHg), mean (SD)	83.3 (12.3)	84.4 (11.4)	83.8 (11.8)
Minimum diastolic blood pressure (mmHg), mean (SD)	42.0 (10.0)	44.1 (11.9)	43.0 (11.0)
Maximum heart rate (beat per min), mean (SD)	95.6 (15.7)	98.5 (11.5)	97.1 (13.7)
Minimum heart rate (beat per min), mean (SD)	67.1 (11.5)	69.4 (11.9)	68.2 (11.7)
Additional PACU analgesia, n (%)^a^			
Paracetamol	3 (5.7)	0	3 (2.8)
Paracetamol/ Fentanyl	0	1 (1.9)	1 (0.9)
Paracetamol/ Mefenamic acid	1 (1.9)	0	1 (0.9)
Duration of surgery (min), mean (SD)	58.7 (20.4)	63.6 (25.4)	61.2 (23.1)
Duration of stay in PACU (min), mean (SD)	78.9 (27.2)	79.2 (26.7)	79.1 (28.8)

*IQR* interquartile range; *LA* local anaesthetics; *PACU* post-anaesthetic care unit; *SD* standard deviation

^a^ Missing data in control group (n = 1)

Music listening was associated with significantly lower postoperative VAS-A scores (MD -1.43, 95%CI -0.63 to -2.22, p < 0.001) (Table 2). Additionally, parturients who received music listening reported significantly lower PCS total scores (MD -6.39, 95%CI -2.11 to -10.66, p = 0.003), as well as rumination (MD -1.68, 95%CI -0.12 to -3.25, p = 0.035), magnification (MD -1.53, 95%CI -0.45 to -2.62, p = 0.006), and helplessness (MD -3.17, 95%CI -1.29 to -5.06, p = 0.001) sub-scores. No significant difference in acute pain scores was detected between parturients who received music listening compared to those who did not. Finally, all parturients who received music listening in the preoperative period (n = 53, 100%) and 46 (86.8%) in the postoperative period were satisfied (rated “excellent” and “good”) with the intervention (Table 3). Parturients’ feedback indicated that music listening was “helpful” (n = 14), “relaxing” or “calming” (n = 9), “helps to focus on the music instead of the surgery” (n = 7), and “reduces anxiety” (n = 3).

**Table 2:** Preoperative and postoperative psychological and pain characteristics

Variables	Preoperative			Postoperative			
	Music group	Control group	P - value	Music group	Control group	Mean Difference (95% CI)	P - value
VAS-A (0–10)	5.1 (2.4)	4.7 (2.6)	0.392	1.4 (1.7)	2.9 (2.4)	-1.43 (-0.63 to -2.22)	< 0.001
Pain score (0–10)	0.1 (0.6)	0.1 (0.7)	0.968	0.5 (1.4)	0.5 (1.5)	− 0.02 (-0.57 to 0.53)	0.947
PCS total score (0–52)	17.2 (13.0)	19.9 (11.8)	0.251	11.9 (10.7)	18.3 (11.6)	-6.39 (-2.11 to -10.66)	0.003
PCS rumination subscale (0–16)	6.5 (5.0)	7.0 (4.3)	0.602	4.7 (4.4)	6.4 (3.8)	-1.68 (-0.12 to -3.25)	0.035
PCS magnification subscale (0–12)	4.2 (3.1)	4.9 (2.7)	0.163	2.8 (2.8)	4.4 (2.9)	-1.53 (-0.45 to -2.62)	0.006
PCS helplessness subscale (0–24)	6.5 (5.5)	8.0 (5.4)	0.154	4.3 (4.4)	7.5 (5.4)	-3.17 (-1.29 to -5.06)	0.001

Data are expressed in mean (SD)

*CI* Confidence interval; *PCS* Pain catastrophizing scale; *SD* standard deviation; *VAS-A* Visual analog scale- anxiety

**Table 3:** Satisfaction scores and preferences in the music listening group (n = 53)

	Preoperative	Postoperative
Patient satisfaction		
Excellent Good Fair Poor Missing data	39 (73.6) 14 (26.4) 0 0 0	35 (66.0) 11 (20.8) 2 (3.8) 0 5 (9.4)
Music Preferences		
Instrumental/soothing English pop songs Disney music Chinese pop songs Malay Indian Religious Others Missing	17 (32.1) 11 (20.8) 8 (15.1) 6 (11.3) 4 (7.5) 1 (1.9) 5 (9.4) 1 (1.9) 0	13 (24.5) 13 (24.5) 6 (11.3) 6 (11.3) 3 (5.8) 1 (1.9) 4 (7.5) 1 (1.9) 6 (11.3)

Data are expressed in n (%)

## Discussion

The results of this randomized controlled trial suggest that perioperative music listening is associated with significant reduction in anxiety and pain catastrophizing following elective cesarean delivery. There was good to excellent satisfaction and positive feedback with perioperative music listening.

Our finding that parturients who received music listening had significantly reduced postoperative anxiety is consistent with the results of two meta-analyses studying music listening during cesarean delivery [5, 17]. Although prior studies mainly conducted in Europe, USA, and Iran have demonstrated that music listening is efficacious in reducing postoperative anxiety after cesarean delivery, these results may not be generalizable to other countries given the important contribution of socioeconomic, cultural, and education status to surgical anxiety [5, 18]. Therefore, our study provides important evidence that parturients undergoing cesarean delivery in Singapore may benefit from perioperative music listening and describes how music listening could be recommended based on patient feedback.

To our knowledge, this is the first study that showed that perioperative music listening was associated with significant reduction in pain catastrophizing including the total scores and all sub-scores on magnification, rumination and helplessness. Pain catastrophizing is characterized by the tendency to magnify the threat, feeling of helplessness, combined with inability to avoid negative thoughts in the context of anticipated or actual pain [19]. Women with elevated pain catastrophizing had greater acute postoperative pain intensity and increased risk of developing post-cesarean persistent pain [11–13]. Additionally, parturients who reported high pain catastrophizing during pregnancy and postpartum periods had more restricted postpartum physical ability at 6 months postpartum [20]. Our finding that music listening may reduce postoperative pain catastrophizing underscores the significant role of emotion and cognition in the pain experience, and suggests that perioperative music listening may be beneficial in improving both psychological and physical states in the context of negative perceived pain experience. Besides, pain catastrophizing could be reduced with the use of cognitive behavioral therapy or physical therapy in other pain conditions (e.g., fibromyalgia), but these treatments are often cost intensive and time consuming [21]. This is especially challenging to be used in parturients undergoing Cesarean delivery in the hospital setting. Future research could be warranted looking into music listening to reduce pain catastrophizing after delivery.

Despite the significant reduction in pain catastrophizing, music listening was not associated with significant reduction in acute pain scores. This finding differed from a meta-analysis conducted by Masoud et al., which showed reduced postoperative pain with exposure to music listening [17]. In contrast, another meta-analysis by Weingarten et al. found no reduction in postoperative pain scores, although they reported that music listening was associated with significantly lower opioid consumption [5]. Nonetheless, it should be noted that analgesic consumption can also be influenced by various factors such as the attitude of healthcare providers, type of analgesia, fear of adverse effects, etc. [11]. Furthermore, both meta-analyses investigated pain scores up to six hours following cesarean delivery, whereas our study evaluated pain scores in the PACU soon after surgery. It is likely that residual effects of spinal anesthesia may have contributed to the low pain scores assessed in both experimental and control groups.

Based on the high satisfaction scores and positive feedback obtained in this study, music listening was well accepted amongst the parturients. This may have been enhanced by allowing parturients to choose their music. Of interest, the diverse genres of music selected by the parturients suggest that a standardized playlist or music selected by a therapist may be limited; patient choice could be more important. Though this could be challenging in previous studies, allowing parturients to select their own music is presently feasible given the availability of streaming services providing an encompassing library of music genres. Allowing parturients the freedom to choose their own music also upholds the principle of patient autonomy and can be empowering.

We acknowledge several limitations to this study. First, we did not continue with longer follow-up and hence did not collect data on persistent pain to determine if the beneficial effects of perioperative music listening persisted, as we believe that exposure to other factors in the ward environment may influence the parturients’ anxiety and pain, hence confounding the results. Second, we only included parturients who were healthy, had no significant co-morbidities, and who were undergoing elective cesarean delivery. Those with significant co-morbidities and undergoing emergency cesarean delivery may experience higher levels of anxiety, perhaps due to the additional uncertainties about their overall outcome. Therefore, our study results may not be generalizable to all obstetric populations. Future studies should examine the efficacy of perioperative music listening on pain and anxiety on these specific obstetric groups.

## Conclusion

Our study found that perioperative music listening was associated with reduction in anxiety and pain catastrophizing following cesarean delivery. With the good patient satisfaction and feedback received, these findings will recommend the use of music listening in the obstetric setting.

## Data Availability

The datasets generated and/or analyzed during this study are not publicly available due to institutional policy on data confidentiality but are available from the corresponding author on reasonable request.

## Abbreviations

ASA: American Society of Anesthesiologists
CI: confidence interval
CONSORT: Consolidated Standards of Reporting Trials
IQR: interquartile range
LA: local anesthetics
MD: mean difference
PACU: post-anesthetic care unit
PCS: pain catastrophizing scale
SD: standard deviation
VAS-A: visual analog scale-anxiety
